# Teriflunomide Attenuates Immunopathological Changes in the Dark Agouti Rat Model of Experimental Autoimmune Encephalomyelitis

**DOI:** 10.3389/fneur.2013.00169

**Published:** 2013-10-30

**Authors:** Garth E. Ringheim, Lan Lee, Lynn Laws-Ricker, Tomas Delohery, Li Liu, Donghui Zhang, Nicholas Colletti, Timothy J. Soos, Kendra Schroeder, Barbara Fanelli, Nian Tian, Christopher W. Arendt, Deborah Iglesias-Bregna, Margaret Petty, Zhongqi Ji, George Qian, Rajula Gaur, Daniel Weinstock, Jean Cavallo, Juventas Telsinskas, Kathleen McMonagle-Strucko

**Affiliations:** ^1^Inflammation and Immunology Translational Development, Celgene Corporation, Summit, NJ, USA; ^2^Tissue Protection and Repair, Sanofi-Genzyme R&D Center, Bridgewater, NJ, USA; ^3^Neuroimmunology, Sanofi, Framingham, MA, USA; ^4^Flow Cytometry, Molecular Biomarkers, Merck Research Labs, Boston, MA, USA; ^5^Clinical Science and Operation, Global Biostatistics and Programming, Sanofi, Bridgewater, NJ, USA; ^6^Bio-Innovation, BioTherapeutics, Sanofi, Cambridge, MA, USA; ^7^Target and Systems Immunology Institute, TSU/Immuno Inflammation, Sanofi, Bridgewater, NJ, USA; ^8^Clinical Science and Operation, Clinical Exploratory Pharmacology, Sanofi, Bridgewater, NJ, USA; ^9^Electrophysiology-Pharmaco EEG, Bioanalysis/Physiology, Lundbeck Research USA, Inc., Paramus, NJ, USA; ^10^Experimental Pharmacology/Neurology, Immuno-Inflammation, Sanofi, Bridgewater, NJ, USA; ^11^Coding Group, Medical Operation, Sanofi, Bridgewater, NJ, USA; ^12^Molecular Histology and Applied Imaging, Proteogenomics/Genetics and Genomics, Sanofi-Genzyme R&D Center, Bridgewater, NJ, USA; ^13^Immunotoxicology and Experimental Pathology, Biologics Toxicology, Biologics Center of Excellence Janssen Research and Development, LLC, Springhouse, PA, USA; ^14^Phage Display, Biologics Discovery, Sanofi, Framingham, MA, USA

**Keywords:** multiple sclerosis, teriflunomide, experimental autoimmune encephalomyelitis, lymphocytes, dihydro-orotate dehydrogenase

## Abstract

Teriflunomide is an oral disease-modifying therapy recently approved in several locations for relapsing-remitting multiple sclerosis. To gain insight into the effects of teriflunomide, immunocyte population changes were measured during progression of experimental autoimmune encephalomyelitis in Dark Agouti rats. Treatment with teriflunomide attenuated levels of spinal cord-infiltrating T cells, natural killer cells, macrophages, and neutrophils. Teriflunomide also mitigated the disease-induced changes in immune cell populations in the blood and spleen suggesting an inhibitory effect on pathogenic immune responses.

## Introduction

Teriflunomide is a novel oral immunomodulatory drug recently approved in several locations as a disease-modifying treatment for relapsing-remitting multiple sclerosis (RRMS) (1). Immune cell activation and proliferation, which are critical for the progression of autoimmune diseases such as MS, are dependent on *de novo* pyrimidine synthesis (2), and extensive *in vitro* studies have shown that teriflunomide is a selective and reversible inhibitor of mitochondrial dihydro-orotate dehydrogenase (DHO-DH) (3), the key enzyme involved in this process (4, 5). Teriflunomide reversibly arrests cells in the G1 phase of the cell cycle (6) and slowly dividing or resting cells, which rely on the salvage pathway for pyrimidine synthesis, are relatively unaffected. Teriflunomide has been shown to have a cytostatic effect on proliferating T and B lymphocytes (7), but further *in vivo* studies are necessary to characterize its therapeutic effects and mechanism of action more fully.

Experimental autoimmune encephalomyelitis (EAE) is a well-studied animal model of human MS (8, 9). EAE is induced by injecting susceptible animals with central nervous system (CNS) extract, purified myelin components, or synthesized specific peptides [derived from myelin oligodendrocyte glycoprotein (MOG), proteolipid protein (PLP), or myelin basic protein (MBP)] emulsified in an adjuvant (10–12). The Dark Agouti (DA) rat model of EAE mimics certain aspects of the clinical course of disease in people with RRMS (9), typified by progressive, sustained demyelination, and associated axonal loss (13–15). In the DA rat model of EAE, neurological impairments, manifesting as a flaccid tail, are observed at disease onset, followed by an acute attack with disturbed gait and paresis/paralysis. Most animals recover from paralysis and experience remission, but then may undergo one or more relapses (10).

The neurological impairment in EAE is mediated by activation of autoimmune responses and is accompanied by infiltration of activated T cells, B cells, natural killer (NK) cells, and monocytes into the affected CNS tissue (10, 16). The development and progression of EAE are reduced by certain immunomodulatory drugs, corticosteroids, cytokines, chemokines, and cells with anti-inflammatory functions, such as regulatory T cells and specific monocyte subtypes (17–23). Teriflunomide has been shown to ameliorate EAE disease severity by reducing inflammation, demyelination, and axonal loss in the cervical spinal cord in the DA rat model (24). However, changes in circulating and tissue-associated specific immune cell subtypes in the presence or absence of teriflunomide have not yet been well characterized.

To gain further insight into the therapeutic effects of teriflunomide treatment, a preclinical study was performed in the DA rat model of EAE. The aim of this study was to characterize the effects of teriflunomide on immune cell numbers and distribution during EAE progression.

## Materials and Methods

### Study design

To measure the effects of teriflunomide on different immune cell populations in the DA rat EAE model, a therapeutic treatment design was used with negative and positive controls. Animals were assigned to undergo EAE induction (*n* = 131) or to remain naïve to immunization and receive vehicle sham treatment (negative control; *n* = 39). Animals that showed signs of EAE were randomly assigned to receive once-daily oral treatment with vehicle (positive control; *n* = 45) or teriflunomide (10 mg/kg) (treatment group; *n* = 45) until study end. Negative control animals received the same daily handling as EAE-induced animals, and received vehicle orally on a once-daily basis.

### Experimental animals and EAE induction

This study was carried out in accordance with an animal use protocol approved by the Institutional Animal Care and Use Committee (25).

Healthy, male, 6-week-old DA rats (Harlan Laboratories, Indianapolis, IN, USA) were housed three to a cage in a room maintained at 23 ± 2°C with a relative humidity of 50% and a 12-h light cycle. They were allowed *ad libitum* access to a commercial diet (Harlan Teklad 2016, Harlan Laboratories, Madison, WI, USA) and filtered water, and were acclimated to the facility for 2–4 weeks prior to study initiation. At study initiation, animals were 8–10 weeks of age and weighed between 162 and 242 g.

Experimental autoimmune encephalomyelitis was induced by immunization with a single 0.2 mL intradermal/subcutaneous injection of an encephalitogenic inoculant emulsion [rat spinal cord homogenate 50% (w/v) in saline mixed with an equal volume of Complete Freund’s Adjuvant containing *Mycobacterium tuberculosis* 7 mg/mL] at the base of the tail (24). Pertussis toxin was not used in the induction of EAE. Beginning 5 days post-inoculation, neurological scores in immunized rats were assessed daily in an unblinded fashion. Clinical disease was scored for typical signs according to the following scale: 0 = normal; 0.5 = partial loss of tail tone; 1 = complete tail atony; 2 = complete tail atony with hindlimb weakness; 2.5 = hindlimbs weak; 3 = hindlimb paresis; 3.5 = hindlimb paresis with one leg completely paralyzed; 4 = complete paralysis; 4.5 = moribund state; 5 = death due to EAE.

### Compound administration

On the first day of disease onset (functional deficit score ≥1), immunized rats received their first dose of teriflunomide [suspended in vehicle: carboxymethylcellulose made up to 0.06% (w/v) in water, to which Tween 80 was added to reach a final concentration of 0.5% (v/v)] or vehicle only. Teriflunomide was formulated daily at a concentration of 10 mg/mL. Rats were dosed by oral gavage at a volume of 1.0 mL/kg. This yielded an effective dose of 10 mg/kg. Negative control (naïve) animals received their first oral dose of vehicle on the same day as corresponding immunized rats. Oral treatment continued once daily for all animals until sacrifice for sampling.

### Sampling

Animals were allocated to one of four groups for sampling, according to EAE stage reached in the positive controls: (1) disease onset (functional deficit score of ≥1); (2) acute attack (score of 3 or 4 for two consecutive days); (3) remission (drop of ≥1 score for two consecutive days); (4) relapse (increase of ≥1 full score from the trough of remission for two consecutive days). Each group contained 10–15 rats. Tissue samples (blood, cervical spinal cord, and spleen) were collected for analysis at each stage. To keep sample selection unbiased, when each positive control (immunized, vehicle-treated) rat reached the required disease stage and was sacrificed, one negative control (naïve, vehicle-treated) rat and one teriflunomide-treated rat were also sacrificed. The negative control group was used for baseline measurements at each disease stage.

On the day of sampling, rats were euthanized with CO_2_ and blood was collected via cardiac puncture. A 600 μL aliquot of whole blood was taken and analyzed on the Advia 120 automated cell counter (Siemens Healthcare Diagnostics, Newark, DE, USA) to determine total cell counts of basophils, eosinophils, lymphocytes, monocytes, neutrophils, and white blood cells (WBCs). Rat peripheral blood mononuclear cells (PBMCs) were isolated from whole blood by Ficoll-Hypaque density gradient centrifugation, using Histopaque^®^ 1077 (Sigma, St. Louis, MO, USA), followed by a red cell lysis procedure using Ammonium-Chloride-Potassium (ACK) lysis buffer. PBMCs were suspended in staining buffer (BD Biosciences, San Jose, CA, USA) and stored at +4°C until antibody staining for flow cytometry.

Spinal cords were fluid-perfused out of excised spinal columns with phosphate-buffered saline (PBS) and the cervical region dissected from the full-length spinal cord. Cells from the spinal cord were isolated by gently grinding the tissue through a 70 μm cell strainer, followed by washing and filtering out debris through a clean cell strainer. Filtered cells were centrifuged and re-suspended in staining buffer at 1 × 10^7^ cells/mL and the samples were stored at +4°C until use.

Spleens were excised and weighed. Splenocytes were isolated as described above for the spinal cord preparations, followed by a red cell lysis procedure using ACK lysis buffer. Cells were re-suspended in staining buffer at 1 × 10^7^ cells/mL and the samples were stored at +4°C until use.

### Histopathological evaluation

#### Tissue and section preparation

A 2 mm cervical spinal cord sample from each animal was immersed in 10% (w/v) buffered formalin for 48 h at +4°C before being transferred into PBS. Samples were processed and embedded into paraffin blocks, each block containing 16–20 cervical cords from different treatment groups. Twenty 5 μm sections were prepared from each block and mounted onto Super Frost^®^ Plus glass slides (Menzel-Gläser, Braunschweig, Germany).

#### Histology and immunohistochemistry

Immunohistochemistry to evaluate inflammatory cell subtypes was performed on the automated Discovery XT instrument, using the DAB Map™ detection kit (Ventana Medical Systems, Tucson, AZ, USA). Overall, inflammatory cell distribution was assessed with hematoxylin and eosin staining (Rowley Biochemical Institute, Inc., Danvers, MA, USA). Demyelination was evaluated with Luxol^®^ fast blue staining (Rowley Biochemical Institute Inc., Danvers, MA, USA). T cells were stained with anti-CD3 antibody (Invitrogen Zymed, San Francisco, CA, USA), and macrophages and microglia were stained with anti-Iba1 antibody (Wako Chemicals USA Inc., Richmond, VA, USA). CD3 and Iba1 antibody-stained slides were scanned with the Aperio scanner (Aperio Technologies, Vista, CA, USA); digital images were analyzed with Aperio algorithms for total number of CD3^+^ cells and total percentage (relative to spinal cord area) of positive staining by anti-Iba1 antibody. Neutrophils were stained with anti-myeloperoxidase antibody (Abcam, Cambridge, MA, USA); myeloperoxidase antibody-stained slides were assessed for staining and then scored visually in a semi-quantitative manner.

### Flow cytometry analysis

To assess lymphocyte composition, 1 × 10^6^ cells were re-suspended in staining buffer and stained for cell-surface markers with fluorescent dye-conjugated antibodies (all from BD Biosciences, San Diego, CA, USA). Cell samples were analyzed on the FACSCalibur^®^(BD, Franklin Lakes, NJ, USA) flow cytometer using Cell Quest™ Pro software version 5.2.

In all samples, purified mouse immunoglobulin (Ig)G1 anti-rat CD32 (clone D34-485) was used (1 μg/million cells) to block non-specific binding to the high-affinity IgG binding receptor, Fc gamma RII. The proportions of CD3^+^, CD4^+^, and CD8^+^ positive T cells in splenocytes, cervical spinal cord cells, and PBMCs were determined by staining cells with mouse IgM anti-rat CD3 allophycocyanin (APC; clone 1F4), mouse IgG2a anti-rat CD4 R-phycoerythrin (PE; clone OX-35), and mouse IgG1 anti-rat CD8α peridinin-chlorophyll (PerCP; clone OX-8). Unstained cells and cells stained with the corresponding isotype fluorescent antibodies mouse IgM APC (clone G155-228), mouse IgG2a PE (clone G155-178), and mouse IgG1 PerCP (clone MOPC-31C) were used as controls for calibrating the instrument and gating positive cells. For analysis of lymphocytes, a canonical “lymphocyte gate,” based on forward and side scatter (FSC/SSC) was drawn to distinguish the lymphocyte population from larger and more granular cells. The lymphocyte gate was adjusted for each tissue type throughout the experiment to adjust for differences in lymphocyte cell size and granularity and to eliminate contaminating debris.

Relative percentages of T cells, B cells, and NK cells within the lymphocyte population in splenocytes, cervical spinal cord, and PBMCs were assessed by staining with a rat T-cell, B-cell, NK-cell (T/B/NK) cocktail: mouse IgM anti-rat CD3 APC (clone 1F4), mouse IgG1 anti-rat CD45RA fluorescein isothiocyanate (FITC; clone OX-33), and mouse IgG1 anti-rat CD161a PE (clone 10/78). Unstained cells and cells stained with the Ig Isotype Control Cocktail – A: mouse IgG1 FITC (clone MOPC-21), mouse IgG1 PE (clone MOPC-21), and mouse IgM APC (clone G155-228) were used as controls for calibrating the instrument and gating positive cells.

### Statistical analysis

Mean and standard error of the mean values were calculated per group at each disease stage for each variable: neurological score; spleen weight; total cell counts in whole blood; and flow cytometry percentage of gated cells. Proportions of CD3^+^ T cells, CD45RA^+^ B cells, and CD161a^+^ NK cells were calculated as a percentage of total lymphocytes. Proportions of CD4^+^ and CD8^+^ T cells were calculated as a percentage of CD3^+^ T cells. Out of 170 animals (131 inoculated and 39 naïve), 21 (12%) had some or all samples not available for flow cytometry analysis.

For each variable at each stage, two comparisons were of interest: (1) negative control (naïive) vs. positive control (EAE-induced), to validate the disease model; (2) positive control vs. teriflunomide, to assess treatment effect. No comparison was made between teriflunomide and negative control groups. Comparisons of interest were conducted using a parametric or non-parametric two-way analysis of variance (ANOVA) with treatment, day, and treatment × day interaction as factors. Specifically, two-way ANOVA with a common variance was used if assumptions regarding normality of distribution and equal variance were satisfied; two-way ANOVA adjusted for unequal variances was used if only the normality assumption was satisfied; and non-parametric two-way ANOVA was used if the normality assumption was not satisfied. Immunohistochemistry data at each disease stage for each endpoint were analyzed using a non-parametric one-way ANOVA model. Analyses were carried out using SAS^®^ version 9.2 (SAS, Cary, NC, USA).

## Results

### Neurological scores and spinal cord analysis

**Figure 1:**
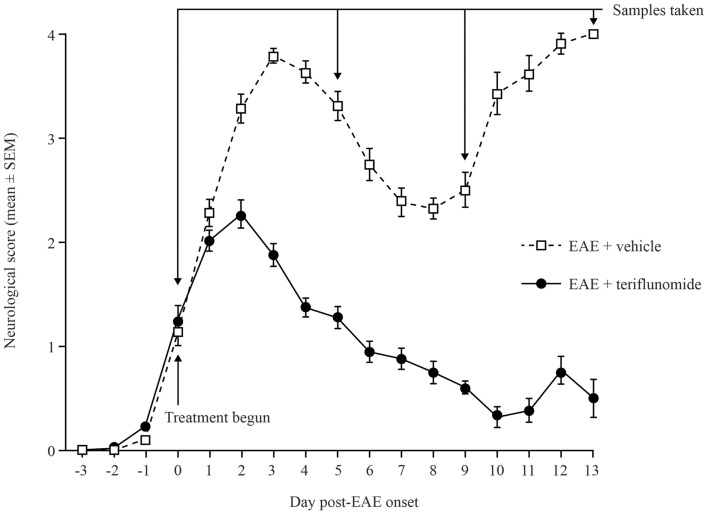
**Neurological deficit scores in teriflunomide-treated and control Dark Agouti EAE rats**. EAE was induced by immunization with spinal cord homogenate. Animals showing signs of EAE were assigned to receive daily oral teriflunomide (10 mg/kg) or vehicle from the first day of disease onset (functional deficit score ≥1, labeled as day 0) throughout the study until sacrifice for sampling, and neurological scores were assessed daily. Cohorts (10–15 animals per treatment group per time point) were sacrificed at onset (day 0), acute attack (day 5), remission (day 9), and relapse (day 13), according to the disease stage of EAE-vehicle-treated rats. Scoring key: 0 = normal; 1 = complete tail atony; 2 = complete tail atony with hindlimb weakness; 3 = hindlimb paresis; 4 = complete paralysis; 5 = death due to EAE. EAE, experimental autoimmune encephalomyelitis; SEM, standard error of the mean.

Dark Agouti rats immunized with spinal cord homogenate developed EAE symptoms with characteristic disease onset, acute attack, remission, and relapse stages. In comparison, rats treated with teriflunomide from the time of disease onset developed reduced levels of neurological deficits vs. vehicle-treated rats, and achieved durable remission (Figure 1).

**Figure 2:**
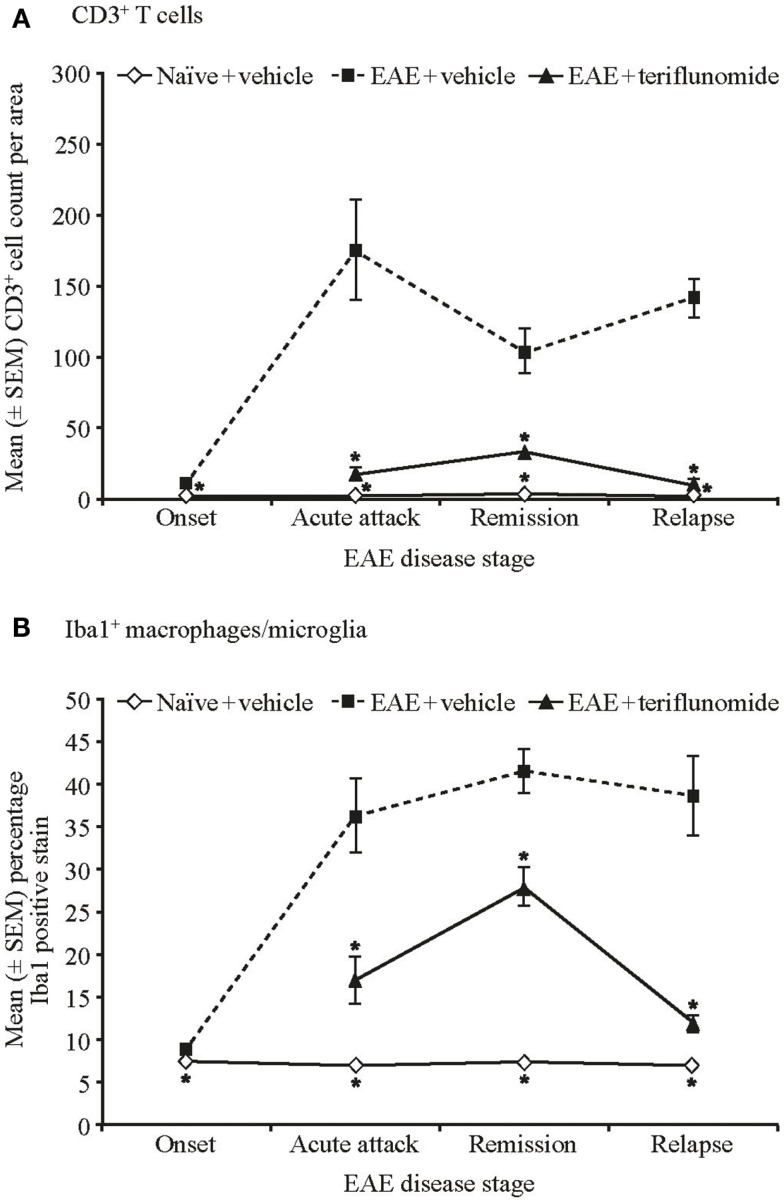
**Numbers of T cells and percent positive staining for microglial cells by immunohistochemistry in cervical spinal cord sections from EAE rats**. Automated digital image analysis of inflammatory cell populations in immunohistochemistry stained tissue sections of cervical spinal cord from untreated and EAE rats (vehicle and teriflunomide-treated) sacrificed at the indicated disease stages (onset, acute attack, remission, relapse). *n* = 5–10 animals per treatment group per disease stage. **(A)** T cells identified with anti-CD3 antibody staining **(B)** microglial cells (macrophages) identified with anti-Iba1 antibody staining. Percent positive staining for microglial cells and cell counts per area for CD3 are shown. *p < 0.005 vs. comparison group (EAE-vehicle vs. untreated; EAE-teriflunomide vs. EAE-vehicle). EAE, experimental autoimmune encephalomyelitis; SEM, standard error of the mean.

**Figure 3:**
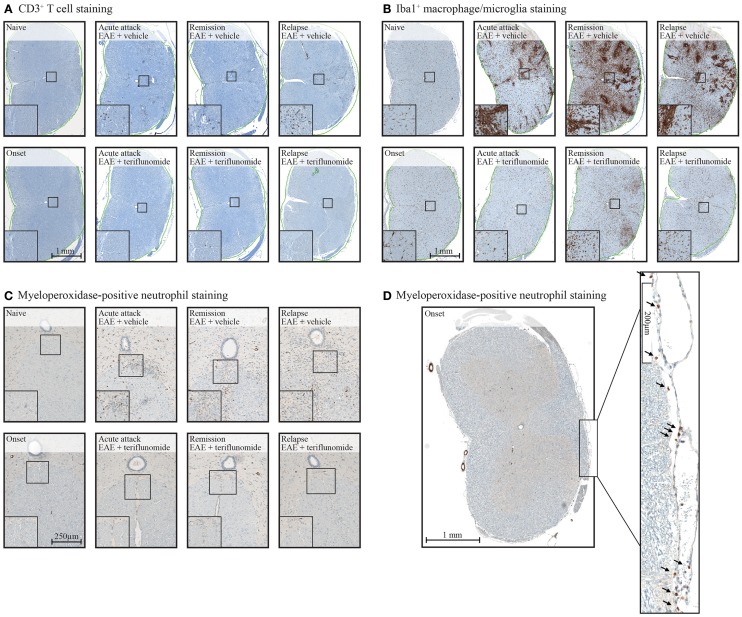
**Staining of CD3^+^ T cells (A), Iba1^+^ macrophages/microglia (B), and myeloperoxidase-positive neutrophils (C,D) in cervical spinal cord sections from EAE rats**. Representative images of immunohistochemistry stained tissue sections used to evaluate inflammatory cell populations in cervical spinal cord from EAE rats (vehicle and teriflunomide-treated) sacrificed at the indicated disease stages (onset, acute attack, remission, relapse). **(A–C)**: upper panels show stained sections of vehicle-treated EAE rats, lower panels show stained sections of teriflunomide-treated EAE rats. Left boxed corner of each panel shows enlargement. Anti-CD3 antibody staining identified T cells **(A)**, anti-Iba1 antibody staining identified microglial cells (macrophages) **(B)**, and myeloperoxidase staining identified neutrophils **(C)**. **(D)** Cervical spinal cord from disease onset in EAE rat stained with myeloperoxidase to identify neutrophils. Arrowheads on the enlarged section of **(D)** indicate neutrophils. EAE, experimental autoimmune encephalomyelitis.

Immunohistochemical analysis of the spinal cord indicated that EAE induction was associated with significant infiltration of CD3^+^ T cells and Iba1^+^ macrophages/microglia at all four disease stages (Figures 2 and 3A,B; Table 1), and with spinal cord infiltration of myeloperoxidase-positive neutrophils at acute attack and relapse (Figure 3C; Table 1). At onset, prior to infiltration and administration of teriflunomide or vehicle, binding of neutrophils to spinal cord endothelium was readily apparent in EAE-induced animals (Figure 3D). Teriflunomide significantly attenuated spinal cord infiltration of CD3^+^ T cells and Iba1^+^ macrophages/microglia at all three treatment phases of the disease (Figure 2; Table 1) and also significantly attenuated increases in myeloperoxidase-positive neutrophils at acute and remission phases (Figure 3C; Table 1).

**Table 1:** **Total number of CD3^+^ T cells, total percentage of Iba1^+^ macrophages/microglia, and myeloperoxidase-positive neutrophils in cervical spinal cord in each treatment group at each disease stage**.

Cell type	Mean (SEM)		
Disease stage	Negative control (naïve + vehicle)	Positive control (EAE + vehicle)	Teriflunomide (EAE + treatment)
**CD3^+^ T cells** (in cervical spinal cord)			
Onset	2.7 (0.3)	10.8 (2.3)^†^	n/a
Acute attack	2.8 (0.4)	175.7 (35.8)^‡^	17.6 (4.9)^‡^
Remission	3.6 (0.4)	104.2 (16.8)^‡^	33.0 (5.3)^‡^
Relapse	2.9 (0.3)	141.9 (13.7)^‡^	9.9 (2.7)**
**Iba1^+^ macrophages/microglia** (in cervical spinal cord)			
Onset	7.4 (0.2)	8.7 (0.5)*	n/a
Acute attack	7.0 (0.2)	36.3 (4.3)^‡^	17.0 (2.8)**
Remission	7.4 (0.1)	41.5 (2.7)^‡^	27.9 (2.3)**
Relapse	6.9 (0.2)	38.5 (4.8)^‡^	11.8 (1.0)^†^
**Myeloperoxidase-positive neutrophils** (in cervical spinal cord)			
Onset	0.3 (0.3)	0.1 (0.1)	n/a
		(EAE, no vehicle)
Acute attack	0 (0)	1.8 (0.4)^‡^	0.3 (0.2)**
Remission	0 (0)	0.5 (0.2)*	0.1 (0.1)
Relapse	0 (0)	2.4 (0.6)^‡^	0 (0)^‡^
**Myeloperoxidase-positive neutrophils** (in blood vessels outside of spinal cord)			
Onset	0.4 (0.3)	1.8 (0.3)**	n/a
		(EAE, no vehicle)
Acute attack	0.2 (0.2)	2.1 (0.4)^‡^	0.8 (0.3)**
Remission	0.1 (0.1)	1.1 (0.3)**	0.2 (0.1)*
Relapse	0 (0)	0.7 (0.2)**	0.6 (0.2)

EAE, experimental autoimmune encephalomyelitis; n/a, not assessed; SEM, standard error of the mean.

n = 5–10 animals per treatment group per disease stage. Immunohistochemistry based scoring data.

*p < 0.05, **p < 0.01, ^†^*p* < 0.001, ^‡^*p* ≤ 0.0001 vs. comparison group (positive control vs. negative control; teriflunomide vs. positive control).

**Figure 4:**
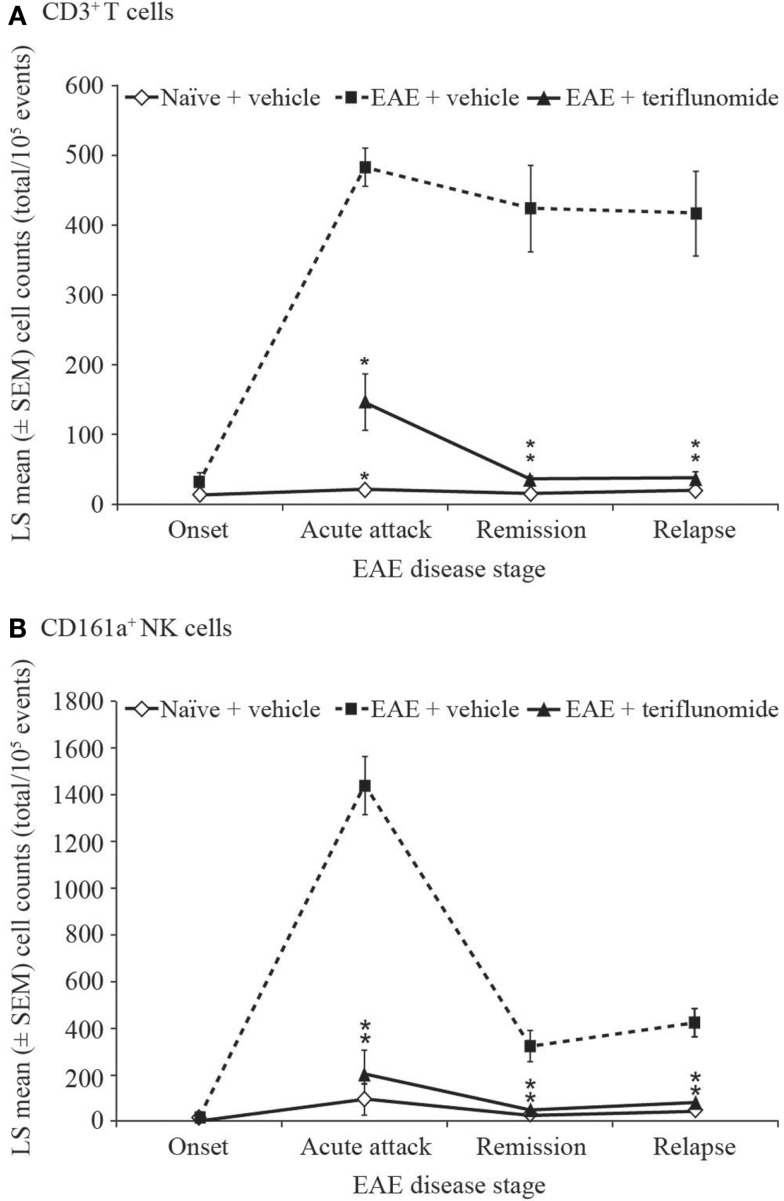
**Number of T cells and NK cells by flow cytometry in cervical spinal cord from EAE rats**. Flow cytometry analysis (cell counts per 10^5^ cells) of immune cell populations in cervical spinal cord from naïve and EAE rats (vehicle and teriflunomide-treated) sacrificed at the indicated disease stages (onset, acute attack, remission, relapse). *n* = 10–15 animals per treatment group per disease stage. **(A)** CD3^+^ T cells and **(B)** CD161a^+^ NK cells were identified by antibody staining. *p ≤ 0.0002 vs. comparison group (EAE-vehicle vs. untreated; EAE-teriflunomide vs. EAE-vehicle). EAE, experimental autoimmune encephalomyelitis; LS, least squares; NK, natural killer; SEM, standard error of the mean.

The inhibitory effect of teriflunomide treatment on the extent of inflammatory immune cell infiltration observed histologically within the spinal cord was also corroborated by flow cytometry analysis of cells isolated from the spinal cord at different stages of disease. As with the immunohistochemical observations, EAE was associated with increases in inflammatory cells, shown by the numbers of CD3^+^ T cells and CD161a^+^ NK cells recovered from the spinal cord at acute attack, remission, and relapse. The increases in both of these cell types were attenuated by teriflunomide (Figure 4).

Overall, these findings indicate a correlation between teriflunomide disease control and inhibition of inflammation within the spinal cord.

### Peripheral changes in immune cell counts

Changes occurring in the periphery during the course of teriflunomide treatment were also investigated in terms of total cell counts and proportions of various immune cell populations. At a gross level, induction of EAE was associated with reductions in spleen weight at acute attack, remission, and relapse; teriflunomide treatment attenuated these weight reductions (Table 2). In whole blood, disease-associated changes in immune cell populations (basophils, eosinophils, lymphocytes, monocytes, neutrophils, and WBCs) were observed; the effects of teriflunomide treatment on these changes are detailed in Table 3.

**Table 2:** **Spleen weights in each treatment group at each disease stage**.

Disease stage	Least-squared mean (SEM) spleen weight (mg)		
	Negative control (naïve + vehicle)	Positive control (EAE + vehicle)	Teriflunomide (EAE + treatment)
Onset	467.4 (21.5)	474.1 (18.5)	n/a
Acute attack	464.8 (11.1)	347.6 (6.1)^‡^	395.0 (9.6)^†^
Remission	513.6 (17.4)	417.4 (10.8)^‡^	472.3 (10.6)**
Relapse	513.0 (9.2)	437.5 (9.7)*	511.2 (23.8)*

EAE, experimental autoimmune encephalomyelitis; n/a, not assessed; SEM, standard error of the mean.

n = 10–15 animals per treatment group per disease stage.

*p < 0.05, **p < 0.01, ^†^*p* < 0.001, ^‡^*p* < 0.0001 vs. comparison group (positive control vs. negative control; teriflunomide vs. positive control).

**Table 3:** **Total cell counts in whole blood in each treatment group at each disease stage**.

Cell type and disease stage	Least-squared mean (SEM) cell count (×10^3^/μL)		
	Negative control (naïve + vehicle)	Positive control (EAE + vehicle)	Teriflunomide (EAE + treatment)
Basophils			
Onset	0.053 (0.013)	0.073 (0.012)	n/a
Acute attack	0.067 (0.005)	0.018 (0.004)^‡^	0.046 (0.005)^†^
Remission	0.053 (0.006)	0.049 (0.005)	0.063 (0.007)
Relapse	0.051 (0.004)	0.058 (0.006)	0.054 (0.004)
Eosinophils			
Onset	0.071 (0.017)	0.106 (0.005)	n/a
Acute attack	0.095 (0.011)	0.041 (0.004)^‡^	0.048 (0.003)
Remission	0.064 (0.009)	0.120 (0.010)^†^	0.070 (0.007)^†^
Relapse	0.102 (0.013)	0.155 (0.011)**	0.082 (0.011)^‡^
Lymphocytes			
Onset	6.626 (1.125)	4.401 (1.182)	n/a
Acute attack	7.613 (0.317)	2.317 (0.194)^‡^	3.525 (0.157)^†^
Remission	6.277 (0.472)	4.397 (0.153)*	3.907 (0.125)*
Relapse	8.583 (0.474)	3.802 (0.275)^‡^	5.709 (0.213)**
Monocytes			
Onset	0.114 (0.038)	0.316 (0.065)	n/a
Acute attack	0.102 (0.010)	0.349 (0.042)^‡^	0.153 (0.016)^‡^
Remission	0.314 (0.147)	0.437 (0.025)	0.146 (0.013)^‡^
Relapse	0.145 (0.008)	0.299 (0.016)^‡^	0.197 (0.021)**
Neutrophils			
Onset	0.996 (0.109)	8.875 (0.590)^‡^	n/a
Acute attack	1.057 (0.084)	6.511 (0.933)**	2.611 (0.261)**
Remission	0.919 (0.072)	6.855 (0.432)^‡^	4.002 (0.438)^‡^
Relapse	1.432 (0.031)	8.050 (0.394)^‡^	5.278 (0.539)**
Total white blood cells			
Onset	8.096 (1.344)	14.074 (1.451)*	n/a
Acute attack	9.029 (0.373)	9.331 (1.041)	6.454 (0.287)*
Remission	7.745 (0.548)	12.002 (0.572)^‡^	8.314 (0.468)^‡^
Relapse	10.423 (0.349)	12.491 (0.521)	11.441 (0.743)

EAE, experimental autoimmune encephalomyelitis; n/a, not assessed; SEM, standard error of the mean.

n = 10–15 animals per treatment group per disease stage. Cell counts were measured using an ADIVA automated cell count system.

*p < 0.05, **p < 0.01, ^†^*p* < 0.001, ^‡^*p* ≤ 0.0001 vs. comparison group (positive control vs. negative control; teriflunomide vs. positive control).

Basophils were reduced at acute attack in EAE-induced animals compared with naïve animals, but were not significantly different at other disease stages. Teriflunomide attenuated the disease-associated basophil elevation at acute attack and had no effect on levels at remission and relapse.

Circulating eosinophil levels showed a biphasic alteration in EAE-induced animals, with a reduction at acute attack followed by elevation during remission and relapse. Teriflunomide did not alter the disease-associated reduction at acute attack, but did attenuate the disease-associated elevations at remission and relapse.

An insignificant trend toward reduced lymphocyte (T- and B-cell) counts in EAE-induced animals occurred at disease onset, progressing to significant reductions at acute attack, remission, and relapse. Teriflunomide significantly attenuated the reductions in circulating lymphocytes at acute attack and relapse. In contrast, at remission, circulating lymphocyte counts showed a greater reduction in the teriflunomide group than in the EAE group.

Circulating monocyte counts were significantly increased at the peak disease stages of acute attack and relapse in EAE-induced animals. Both stages of elevations were attenuated by teriflunomide. At remission, monocyte counts were similar in EAE and naïve animals, but were significantly lower in the teriflunomide-treated animals.

Disease-associated elevations in circulating neutrophils were observed at all four stages of disease. Following disease onset, when treatment was initiated, disease-associated neutrophil elevations were significantly attenuated by teriflunomide.

Total WBC counts were elevated in EAE-induced animals vs. naïve animals at disease onset and remission, with no significant change at acute attack or relapse. Teriflunomide treatment had an early effect of reducing disease-associated elevations in WBCs at acute attack and remission disease stages, but did not have an effect at the later relapse stage.

Overall, teriflunomide treatment tended to normalize immune cell changes induced by the disease process.

### Peripheral immune cell population dynamics

The impact of teriflunomide treatment on immune cell population dynamics in the spleen and blood was analyzed by flow cytometry. In the spleen, reductions in CD3^+^ T cells (as a percentage of total lymphocyte populations, measured by two independent antibody detection methods) occurred in EAE-induced animals at disease onset, acute attack, and relapse (Tables 4 and 5). A reduction was also observed at remission, reaching significance in the T-cell panel (Table 4) although not in the T/B/NK panel (Table 5). In both assay methods, teriflunomide showed a non-significant trend of attenuating disease-associated T-cell reductions at acute attack and remission, and a significant attenuation at relapse (Tables 4 and 5). Increases in the percentage of CD4^+^ T cells and decreases in the percentage of CD8^+^ T cells were observed in EAE-induced animals at all four disease stages. Teriflunomide attenuated these changes at acute attack and remission, but not at relapse (Table 4).

**Table 4:** **Proportion^a^ of CD3^+^, CD4^+^, and CD8^+^ T cells in spleen in each treatment group at each disease stage**.

Cell type	Least-squared mean (SEM) percentage		
Disease stage	Negative control (naïve + vehicle)	Positive control (EAE + vehicle)	Teriflunomide (EAE + treatment)
CD3^+^ T cells			
Onset	42.38 (0.92)	37.92 (1.00)*	n/a
Acute attack	46.55 (1.32)	40.13 (2.01)**	42.72 (1.29)
Remission	50.35 (0.30)	45.01 (1.45)**	47.91 (1.14)
Relapse	50.01 (1.03)	34.11 (2.00)^‡^	43.40 (1.28)^†^
CD4^+^ T cells			
Onset	62.19 (1.92)	70.24 (0.76)^†^	n/a
Acute attack	70.96 (1.23)	77.92 (0.88)^‡^	75.56 (0.48)*
Remission	72.94 (0.44)	80.06 (0.64)^‡^	75.87 (0.58)^‡^
Relapse	71.53 (0.67)	75.23 (1.00)^†^	75.21 (0.51)
CD8^+^ T cells			
Onset	32.57 (0.74)	26.67 (0.77)**	n/a
Acute attack	26.17 (1.11)	19.17 (0.72)^†^	21.57 (0.47)*
Remission	24.26 (0.41)	17.26 (0.65)^‡^	21.25 (0.57)^§^
Relapse	24.88 (0.42)	20.79 (0.59)^‡^	21.88 (0.44)

EAE, experimental autoimmune encephalomyelitis; n/a, not assessed; SEM, standard error of the mean.

n = 10–15 animals per treatment group per disease stage. Cell frequencies were measured using flow cytometry.

^a^CD3^+^ T cells as a percentage of total lymphocytes; CD4^+^, and CD8^+^ T cells as a percentage of CD3^+^ T cells.

*p < 0.05, **p < 0.01, ^†^*p* < 0.001, ^‡^*p* < 0.0001 vs. comparison group (positive control vs. negative control; teriflunomide vs. positive control).

**Table 5:** **Proportion^a^ of CD3^+^ T cells, CD45RA^+^ B cells, and CD161a^+^ NK cells in spleen in each treatment group at each disease stage**.

Cell type	Least-squared mean (SEM) percentage		
Disease stage	Negative control (naïve + vehicle)	Positive control (EAE + vehicle)	Teriflunomide (EAE + treatment)
CD3^+^ T cells			
Onset	35.78 (1.13)	31.03 (1.86)*	n/a
Acute attack	35.55 (1.40)	31.07 (1.80)*	34.09 (1.16)
Remission	42.56 (2.32)	39.18 (1.39)	42.08 (1.85)
Relapse	43.23 (1.12)	32.85 (1.69)^‡^	43.32 (0.97)^‡^
CD45RA^+^ B cells			
Onset	37.01 (0.51)	36.08 (1.33)	n/a
Acute attack	38.15 (0.72)	33.04 (1.01)^‡^	36.79 (1.01)**
Remission	36.00 (1.68)	32.12 (1.15)	27.50 (0.77)**
Relapse	35.00 (0.56)	24.76 (0.57)^‡^	29.56 (1.25)*
CD161a^+^ NK cells			
Onset	11.46 (0.47)	13.09 (0.30)	n/a
Acute attack	12.47 (0.49)	15.76 (0.36)^†^	13.19 (0.76)**
Remission	9.52 (0.33)	15.82 (0.77)^‡^	16.41 (0.68)
Relapse	10.56 (0.49)	26.82 (1.60)^‡^	14.23 (0.81)^‡^

EAE, experimental autoimmune encephalomyelitis; n/a, not assessed; NK, natural killer; SEM, standard error of the mean. Cell frequencies were measured using flow cytometryn = 10–15 animals per treatment group per disease stage.

^a^All cell types as a percentage of total lymphocytes.

*p < 0.05, **p < 0.01, ^†^*p* < 0.001, ^‡^*p* ≤ 0.0001 vs. comparison group (positive control vs. negative control; teriflunomide vs. positive control).

Induction of EAE was associated with significant reductions in CD45RA^+^ B cells (as a percentage of total lymphocytes) at acute attack and relapse, with a non-significant decrease observed at remission. Disease-associated reductions in CD45RA^+^ B cells were attenuated by teriflunomide at acute attack and relapse, whereas the proportion of CD45RA^+^ B cells was lower in the teriflunomide group than in the EAE group at remission (Table 5).

Percentages of CD161a^+^ NK cells were increased at acute attack, remission, and relapse in EAE-induced animals; teriflunomide attenuated the observed increases at acute attack and relapse, but not at remission (Table 5).

**Figure 5:**
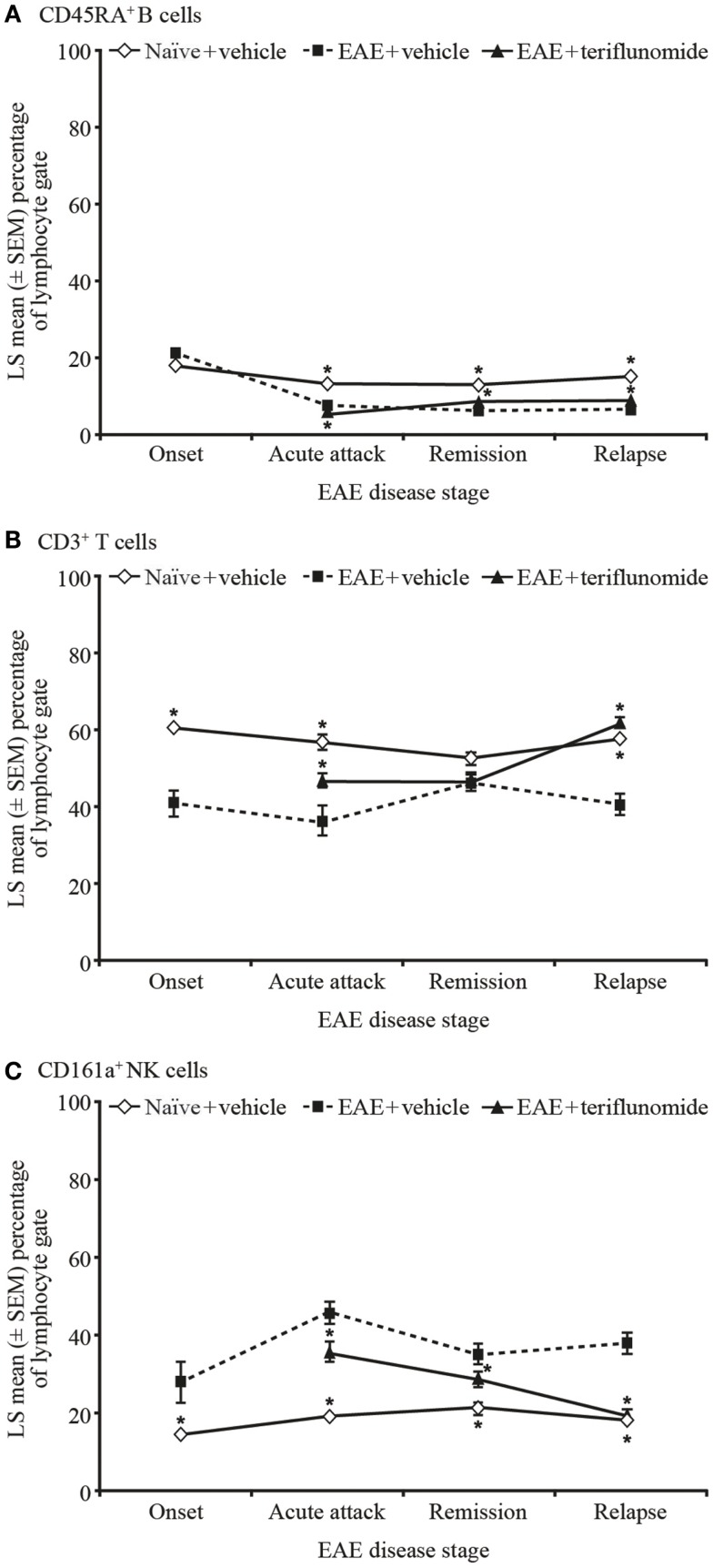
**Proportion of circulating B, T, and NK cells by flow cytometry in PBMCs from EAE rats**. Flow cytometry analysis of immune cell populations in PBMCs from blood taken from naïve and EAE rats (vehicle and teriflunomide-treated) sacrificed at the indicated disease stages (onset, acute attack, remission, relapse). *n* = 10–15 animals per treatment group per disease stage. CD45RA^+^ B cells **(A)**, CD3^+^ T cells **(B)**, and CD161a^+^ NK cells **(C)** were identified by staining with an anti-rat T/B/NK-cell antibody cocktail, and are shown as a percentage of total lymphocytes. *p < 0.05 vs. comparison group (EAE-vehicle vs. untreated; EAE-teriflunomide vs. EAE-vehicle). EAE, experimental autoimmune encephalomyelitis; LS, least squares; NK, natural killer; PBMCs, peripheral blood mononuclear cells; SEM, standard error of the mean.

In PBMCs, in the T-cell subset and T/B/NK panel, disease-associated reductions in the percentage of CD3^+^ T cells were seen at onset, acute attack, and relapse, but not at remission. Teriflunomide attenuated the reductions at acute attack and relapse (Figure 5B; Table 6). Increases in the percentage of CD4^+^ T cells, and decreases in the percentage of CD8^+^ T cells, were observed at all four disease stages. Teriflunomide attenuated these changes at remission only (Table 6).

**Table 6:** **Proportion^a^ of CD3^+^, CD4^+^, and CD8^+^ T cells, CD45RA^+^ B cells, and CD161a^+^ NK cells in PBMCs in each treatment group at each disease stage**.

Cell type	Least-squared mean (SEM) percentage		
Disease stage	Negative control (naïve + vehicle)	Positive control (EAE + vehicle)	Teriflunomide (EAE + treatment)
CD3^+^ T cells			
Onset	58.03 (0.85)	40.67 (3.79)^†^	n/a
Acute attack	60.37 (1.59)	38.84 (5.67)^‡^	49.38 (1.91)*
Remission	57.69 (1.90)	52.99 (2.30)	54.75 (2.30)
Relapse	59.73 (0.83)	40.71 (2.95)^‡^	60.84 (1.53)^‡^
CD4^+^ T cells			
Onset	71.19 (0.93)	76.13 (1.24)^†^	n/a
Acute attack	75.12 (0.63)	80.02 (1.01)^‡^	78.84 (0.57)
Remission	76.10 (0.77)	81.59 (0.27)^‡^	77.80 (0.64)^‡^
Relapse	73.07 (0.22)	75.72 (0.57)**	76.14 (0.28)
CD8^+^ T cells
Onset	24.78 (0.43)	19.90 (0.35)^‡^	n/a
Acute attack	21.86 (0.52)	16.42 (0.90)^‡^	16.93 (0.74)
Remission	20.69 (0.67)	15.85 (0.30)^‡^	19.00 (0.66)^‡^
Relapse	24.19 (0.23)	21.40 (0.55)**	21.82 (0.25)
CD45RA^+^ B cells			
Onset	18.03 (0.96)	21.39 (0.79)	n/a
Acute attack	13.06 (0.66)	7.40 (0.52)^‡^	5.72 (0.40)*
Remission	12.90 (0.94)	6.26 (0.49)^‡^	8.47 (0.52)**
Relapse	15.13 (0.47)	6.47 (0.54)^‡^	8.43 (0.54)*
CD161a^+^ NK cells			
Onset	14.10 (1.12)	27.79 (5.46)*	n/a
Acute attack	18.80 (1.08)	45.72 (3.03)^‡^	35.47 (2.65)**
Remission	21.12 (1.35)	35.00 (2.65)^†^	28.52 (2.05)*
Relapse	17.66 (1.24)	37.89 (3.03)^‡^	19.18 (1.43)^‡^

EAE, experimental autoimmune encephalomyelitis; n/a, not assessed; NK, natural killer; PBMCs, peripheral blood mononuclear cells; SEM, standard error of the mean. Cell frequencies were measured using flow cytometry.

n = 10–15 animals per treatment group per disease stage.

^a^CD3^+^ T cells, CD45RA^+^ B cells, and CD161a^+^ NK cells as a percentage of total lymphocytes; CD4^+^ and CD8^+^ T cells as a percentage of CD3^+^ T cells.

*p < 0.05, **p < 0.01, ^†^*p* < 0.001, ^‡^*p* ≤ 0.0001 vs. comparison group (positive control vs. negative control; teriflunomide vs. positive control).

Induction of EAE was associated with reductions in the proportions of CD45RA^+^ B cells at acute attack, remission, and relapse. Teriflunomide treatment reduced B-cell levels further at acute attack, but attenuated disease-associated reductions at remission and relapse (Figure 5A). The percentage of CD161a^+^ NK cells was increased at all four disease stages, and teriflunomide attenuated the increases at all three treatment stages of acute attack, remission, and relapse (Figure 5C).

## Discussion

In this study, we profiled immunological changes in a rat model of RRMS, focusing on immunocytes in the cervical spinal cord, circulation, and spleen during early onset, acute attack, remission, and relapse disease stages. To improve our understanding of the impact that teriflunomide may have on inflammatory disease processes in patients with MS, we further investigated how therapeutic treatment with teriflunomide, beginning at disease onset, affected immunocyte profiles during EAE disease progression. To our knowledge, this preclinical study is the first to examine the effect of teriflunomide on peripheral and CNS immune cell population changes during acute attack, remission, and relapse in the DA rat model of EAE.

The immunological observations in the DA rat model of EAE correlate with known immunopathological changes in MS (10, 24, 26). In this model, spinal cord infiltration of inflammatory effector cells (22, 27), elevation of CD4^+^ T cells and reduction in CD8^+^ T cells in the spleen (18, 20, 28), and early resistance to apoptosis of invading cells (28) may all be key mediators of disease onset and progression. Early neutrophil involvement has also been reported as necessary for EAE progression (29–31). Similarly, MS development and progression are thought to involve immunological changes leading to inflammatory demyelination and axonal loss (13), with putative pathophysiological roles for mitochondrial dysfunction, T-cell infiltration into the CNS, and resistance to apoptosis (14). Moreover, patients with MS have high numbers of neutrophils in a primed state compared with subjects without MS (32, 33).

As expected from earlier studies with DA rats (10, 24), EAE induction was associated with the onset of progressive neurological impairment and influx into the spinal cord of T cells and macrophages and, as shown in this report, NK cells and neutrophils. This association was most evident at the early onset and acute attack disease stages. However, during the remission and relapse stages (when neurological scores changed), numbers of T cells, macrophages, and neutrophils remained elevated, while NK-cell levels declined and remained low thereafter. This suggests that behavioral deficits do not correlate strictly with immunocyte numbers in the spinal cord at the later relapsing-remitting stages of the disease; rather, functional or cell subtype changes within specific immunocyte populations likely play a more dominant role in the observed behavioral and neurological pathology. Such varying roles within immune cell types for both the induction and inhibition of EAE and MS have been described for T cells (34), macrophages (35–37), and NK cells (38, 39).

Peripheral immunocyte population changes during EAE in the DA rat have not been reported previously, therefore changes in spleen and peripheral blood samples were assessed in the present study. Spleen weights were unchanged at early onset, but were significantly decreased at acute attack, remission, and relapse, when active behavioral deficits occurred. Changes in immunocyte composition during EAE progression in the spleen and blood were strikingly similar. T cells as a percentage of total lymphocytes were reduced, with a shift toward higher CD4^+^ and reduced CD8^+^ percentages at all four disease stages. NK cells as a percentage of total cells were also elevated at all four disease stages, whereas B cells were reduced at the most active disease phases of acute attack and relapse. These changes in spleen and blood immunocyte composition likely reflect mobilization of immunocytes during disease, and although assessment of changes in activation state and trafficking patterns were beyond the scope of the present study, changes in T-cell activation, and trafficking patterns in spleen and blood, have been reported in a monophasic EAE rat model (40). As mentioned, through its inhibition of DHO-DH, teriflunomide exerts a cytostatic effect on stimulated lymphocytes (7). In a study of human PBMCs stimulated *in vitro*, Li et al. showed that teriflunomide principally affected proliferation of lymphocytes, with little effect on function (41), and teriflunomide-treated patients have been shown to mount effective responses to seasonal influenza vaccination (42). In an adoptive transfer model of EAE, *in vitro* teriflunomide treatment of MBP-specific T cells prior to transfer reduced maximal disease scores in recipient naïve animals. *In vitro* testing of the same teriflunomide-treated MBP-specific T cells showed defects in antigen-induced proliferation, but only modest functional impairment (43). These studies indicate that teriflunomide-treated cells retain functionality *in vivo*. Therefore, we might expect that the changes we observed in lymphocyte populations in teriflunomide-treated animals arise as a consequence of diminished proliferation, rather than impaired activation. Confirmatory studies to this effect would be of great interest.

Teriflunomide treatment, initiated at the time of disease onset, suppressed neurological deficit progression in EAE-induced DA rats. Other studies in animal models have shown that teriflunomide can also ameliorate or prevent the emergence of symptoms consistent with MS when animals are dosed at remission or prophylactically (24, 44). Studies in patients have also shown therapeutic benefits for patients with relapsing forms of MS, including subgroups with both higher and lower disease activity (45, 46). Taken together, this suggests that teriflunomide is effective whenever it is administered in a relapsing disease course, though future studies in animal models may contribute to an understanding of the optimal point in disease course to begin teriflunomide treatment. The improvement in neurological deficits we observed during teriflunomide treatment was associated with reduced levels of spinal cord-infiltrating T cells, NK cells, macrophages, and neutrophils, and with attenuation of disease-associated changes in circulating lymphocytes, NK cells, and neutrophils. Teriflunomide also attenuated the EAE-associated reduction in spleen weight, elevations of circulating monocytes at acute attack and relapse, and increased ratios of CD4^+^/CD8^+^ T cells in the spleen at acute attack and remission. These treatment effects of teriflunomide, administered after disease onset, suggest that its therapeutic activity may be due to an inhibitory effect on immune cell proliferation and recruitment and, potentially, on transmigration.

Reductions in T-cell, NK-cell, and neutrophil spinal cord infiltration may be partly responsible for the corresponding neurological benefits of teriflunomide treatment. Teriflunomide treatment significantly reduced the numbers of T cells and NK cells infiltrating the cervical spinal cord at all stages of EAE progression, with the lowest levels observed at remission and relapse. Reduced infiltration of T cells into the spinal cord has been shown to suppress EAE induction in mice (47). Notably, a higher CD4^+^/CD8^+^ T-cell ratio in the spinal cord may underlie enhanced EAE susceptibility in mice (48), and enhanced infiltration of NK cells into the spinal cord may exacerbate EAE symptoms in DA rats (49). As the observed reductions in T-cell, NK-cell, and neutrophil spinal cord infiltration and neurological impairment progression occurred after early effector-phase activation, teriflunomide may potentially act to reduce the numbers of cells migrating to the CNS and/or inhibit local expansion of infiltrating immunocytes. Teriflunomide’s effects on the proliferation of activated lymphocytes are expected to lead to decreased CNS inflammation (50). T-cell expansion within the CNS has been documented in the EAE model (51), so it will be important for future studies to establish whether teriflunomide affects the proliferation of T cells within the CNS in addition to its effects on T-cell proliferation in the periphery (41). Other immunomodulatory effects independent of pyrimidine synthesis that have been described for teriflunomide may also account for the reduction in DA rat spinal cord immunocytes observed in this study, including reduced T-cell chemotaxis (43), inhibition of protein aggregation (52), and weakening of T-cell interactions with antigen-presenting cells (53).

The prevention by teriflunomide of neutrophil spinal cord infiltration may account for some of its therapeutic behavioral benefit. Neutrophils, which are early responders to tissue damage, infiltrate the spinal cord early during the acute attack phase in EAE (29) and are implicated in EAE disease progression (30, 31). They also contribute to early inflammatory processes important in EAE pathology, such as the recruitment of CD4^+^ T cells (54), CD8^+^ effector T-cell generation (55), and infiltration of inflammatory monocytes (31). Support for the role of neutrophils in EAE has been demonstrated by selective depletion of Gr-1^+^ neutrophils with antibodies (30). The effects of these antibody treatments appear to be specific for neutrophil depletion because susceptibility to EAE can be restored by injection of purified neutrophils (56). A role for neutrophils in upstream regulation of macrophage infiltration is further suggested by the effects of injecting activated neutrophil secretion products into tissues of neutrophil-depleted animals, which reverses the block in macrophage infiltration (31). Our data in the DA rat EAE model demonstrated that teriflunomide treatment only partially reversed the increases in circulating neutrophils at acute attack and remission (but not at relapse), while spinal cord levels remained low at all disease stages. Thus, the reduced number of circulating neutrophils cannot completely account for the teriflunomide-associated reduction in spinal cord neutrophils, indicating that teriflunomide may also reduce neutrophil infiltration as part of its mechanism of action in attenuating EAE.

Based on the results of this and other studies in the DA rat model of EAE, teriflunomide has several potential CNS benefits in MS. Teriflunomide may decrease infiltration of T and B cells into the spinal cord (via effects on proliferation and potentially on migration), reduce demyelination and axonal damage/loss in the cervical spinal cord, reduce neurodegeneration, and thereby preserve conduction (24). These effects are consistent with the clinical benefits of teriflunomide treatment observed in patients with RRMS (45). In the Teriflunomide Multiple Sclerosis Oral (TEMSO) trial, the first pivotal Phase III study to report findings from the teriflunomide clinical development program, once-daily oral teriflunomide 14 mg significantly reduced annualized relapse rate by over 30% (*p* < 0.001 vs. placebo) and reduced risk of 12-week confirmed disability progression by 30% (*p* = 0.03 vs. placebo) (45). The incidence of serious infections and malignancies in teriflunomide groups was low and comparable to those in the placebo group (45), suggesting that teriflunomide does not cause generalized immunosuppression or lymphocyte toxicity. Indeed, during prolonged teriflunomide exposure, the risk of opportunistic infections is not increased and the incidence of malignancies is comparable to that of the general population (57).

The results of this study both support and further characterize the effects of teriflunomide on the immune system. Changes in immunocyte populations during disease progression in the DA rat EAE model were attenuated by teriflunomide, suggesting its clinical benefits in MS may be achieved via a modulatory effect on a previously activated immune system. Teriflunomide-treated animals remained in remission, demonstrating that clinical benefits may be achieved without complete negation of effector-cell activation. Further information is needed on the relative significance of immunocyte populations and their specific functional impact on the EAE model at different disease stages, and how specific effects of treatment on these populations translate into clinical benefits, in order to continue to build on our understanding of the therapeutic action of teriflunomide.

## Conflict of Interest Statement

Garth E. Ringheim was an employe of Sanofi during this study; he is currently employed at Celgene Corporation. The following authors are employes of Sanofi: Lan Lee, Lynn Laws-Ricker, Li Liu, Donghui Zhang, Nicholas Colletti, Timothy J. Soos, Kendra Schroeder, Nian Tian, Christopher W. Arendt, Zhongqi Ji, George Qian, Rajula Gaur, Jean Cavallo, and Juventas Telsinskas. The following authors were employes of Sanofi at the time the study was conducted: Barbara Fanelli, Tomas Delohery (currently employed at Merck Research Labs, Boston, MA, USA), Deborah Iglesias-Bregna (currently employed at Lundbeck Research USA Inc.), Margaret Petty, Daniel Weinstock (currently employed at Janssen Research and Development, LLC), and Kathleen McMonagle-Strucko.
